# Inferring the effect of interventions on COVID-19 transmission networks

**DOI:** 10.1038/s41598-021-01407-y

**Published:** 2021-11-09

**Authors:** Simon Syga, Diana David-Rus, Yannik Schälte, Haralampos Hatzikirou, Andreas Deutsch

**Affiliations:** 1 Center for Information Services and High Performance Computing, Technische Universität Dresden, Nöthnitzer Straße 46, 01062 Dresden, Germany; 2 Bavarian Health and Food Safety State Authority (LGL), Veterinärstraße 2, 85764 Oberschleißheim, Germany; 3 Institute of Computational Biology, Helmholtz Zentrum München – German Research Center for Environmental Health, 85764 Neuherberg, Germany; 4 Center for Mathematics, Technische Universität München, 85748 Garching, Germany; 5 Mathematics Department, Khalifa University, P.O. Box 127788, Abu Dhabi, UAE

**Keywords:** Viral infection, Complex networks, Statistical physics

## Abstract

Countries around the world implement nonpharmaceutical interventions (NPIs) to mitigate the spread of COVID-19. Design of efficient NPIs requires identification of the structure of the disease transmission network. We here identify the key parameters of the COVID-19 transmission network for time periods before, during, and after the application of strict NPIs for the first wave of COVID-19 infections in Germany combining Bayesian parameter inference with an agent-based epidemiological model. We assume a Watts–Strogatz small-world network which allows to distinguish contacts within clustered cliques and unclustered, random contacts in the population, which have been shown to be crucial in sustaining the epidemic. In contrast to other works, which use coarse-grained network structures from anonymized data, like cell phone data, we consider the contacts of individual agents explicitly. We show that NPIs drastically reduced random contacts in the transmission network, increased network clustering, and resulted in a previously unappreciated transition from an exponential to a constant regime of new cases. In this regime, the disease spreads like a wave with a finite wave speed that depends on the number of contacts in a nonlinear fashion, which we can predict by mean field theory.

## Introduction

The SARS-CoV-2 pandemic has dramatic consequences at a global scale. Until herd immunity has been reached through vaccination, countries rely on non-pharmaceutical interventions (NPIs) of varying severity, like canceling big events, closing schools, and shutting down businesses to reduce virus transmission. An important goal of NPI design is to prevent those contacts in the population which contribute the most to disease spread while allowing less dangerous contacts. Since the start of the pandemic, mathematical models have been playing an important role in guiding policy makers by developing scenarios for the number of cases, hospitalizations and deaths^1,2^, and estimating the effects of NPIs on the spreading dynamics^3–6^. They are also used to estimate the herd immunity threshold^7^. However, the effect of NPIs on the transmission network of the disease and the corresponding changes in the spreading dynamics are still not fully understood.

**Figure 1:**
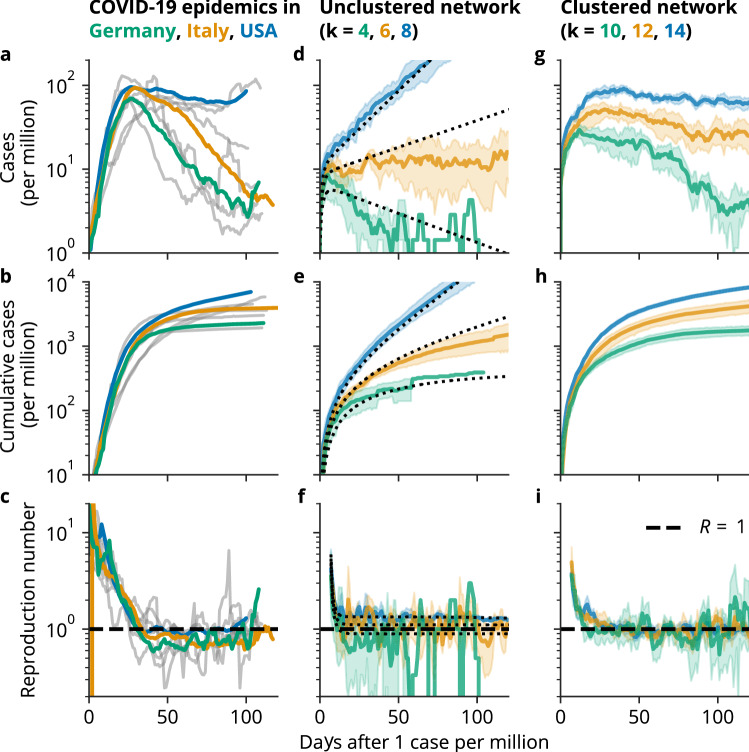
Dynamics of COVID-19 epidemics compared to model dynamics in hypothetical clustered and unclustered networks. (**a–c**) Seven-day rolling average of new cases per million, cumulative cases per million and reproduction number in Germany, Italy and the US, aligned to the time point when the 7-day rolling average reached one per million. After the implementation of NPIs, case numbers decrease slowly, corresponding to effective reproduction numbers of just below one. As an extreme example, in the US, case counts stayed almost constant for approximately 2 months, before increasing again. In contrast, in Germany, cases decreased by about 90% in 2 months, similar to other Western European countries (gray lines). Case counts in Italy, the epicenter of the first wave, are between those of the US and Germany. (**d–f**) Disease dynamics in a random network. The number of new cases and cumulative cases changes exponentially over time, strongly depending on the number of contacts and the infection probability. The reproduction number is equal to one only for a fine-tuned set of parameters. Black dotted lines correspond to predictions of the respective differential equation approximation. (**g–i**) Disease dynamics in a strongly clustered small-world network ($p \approx 0$). After an initial exponential increase in cases, the number of new cases is almost constant over time, corresponding to a linear increase in cumulative cases and reproduction numbers around one. This behavior is robust against changes in the total number of contacts *k* and the infection probability $p_I$. (**d–i**) show mean and standard deviation of 5 independent simulations per parameter set on Watts–Strogatz networks with $n = 10^5$ nodes. The data in (**a–c**) is provided by Johns Hopkins University^28^.

In several countries, following the implementations of various NPIs, the curves of cumulative cases left the exponential regime and entered an approximately linear one, corresponding to a constant number of daily new infections, and an effective reproduction number around one, see Fig. 1a–c. This is remarkable, because these countries are heterogeneous regarding their demographics, economic situation and the implemented NPIs. Epidemiological models that assume a fully connected transmission network, like compartment models based on ordinary differential equations (ODEs), predict this behavior only for a particularly fine-tuned set of parameters, which contrasts with the robustness of the decline in the reproduction number observed in reality^8,9^, see Fig. 1d–f. This is still true for detailed compartment models that incorporate the effect of test–trace–isolate (TTI) efforts, asymptomatic spreaders, or age-dependent spreading based on contact matrices^4,10^ and for agent-based models that rely on coarse-grained contact networks, for example created from anonymized cell phone data, which assume a well-mixed situation on a mesoscopic scale of hundreds of agents^5,11,12^. Komarova et al.^13^ discussed the power law behavior of the dynamics of COVID-19 spread in the context of a metapopulation model, where the population is divided into connected patches assuming a fully connected transmission network within individual patches. The power law dynamics during the hard lockdown in Chinese provinces could be explained by a compartment model that assumes that the susceptible population was quarantined on a time scale comparable to the infectious period of the disease, so that the epidemic comes to a halt quickly^14^. However, this assumption is hard to justify for the situation in Western countries like Germany, that did not implement a full lockdown but enacted contact restrictions and closure of nonessential businesses so that people were still allowed to leave their homes and meet in small groups.

In contrast to models that implicitly assume fully connected transmission networks, network-based epidemiological models allow to consider the effects of heterogeneity with respect to the type and frequency of contacts in the population, i.e. how often people meet and whom, by representing all agents as nodes of a network and the contacts in the population by links between these nodes^15,16^. The heterogeneous topology of real social networks is reflected by a small average path length between any two nodes (small world property), a high clustering in the network (the probability of two nodes being connected is much larger if they have a neighbor in common) and by a power-law distribution (scale-free property) of the node degree^17,18^. The structure of the transmission network should be considered in mathematical models, because it can change the observed spreading dynamics^19^. For example, the spread of diseases is strengthened on scale-free networks so that the epidemic threshold is reduced^20–22^. Thurner et al.^23^ suggested that the linear regime of cumulative cases is a consequence of small-world transmission networks with high clustering, see Fig. 1g–i. A similar system was studied in a theoretical work on epidemic spread with two levels of mixing^24^. There, the authors found that transmission within interconnected cliques leads to an increase in the effective reproduction number proportional to the number of infected people in the cliques. This in turn means that a very small number of links between cliques is enough to enable a large outbreak, which corresponds to the exponential regime. A model based on a spatial transmission network with a variable interaction range also showed power-law growth of new cases^25^. A related model could explain the disease dynamics during the SARS outbreak in Hong Kong in 2003^26^.

**Figure 2:**
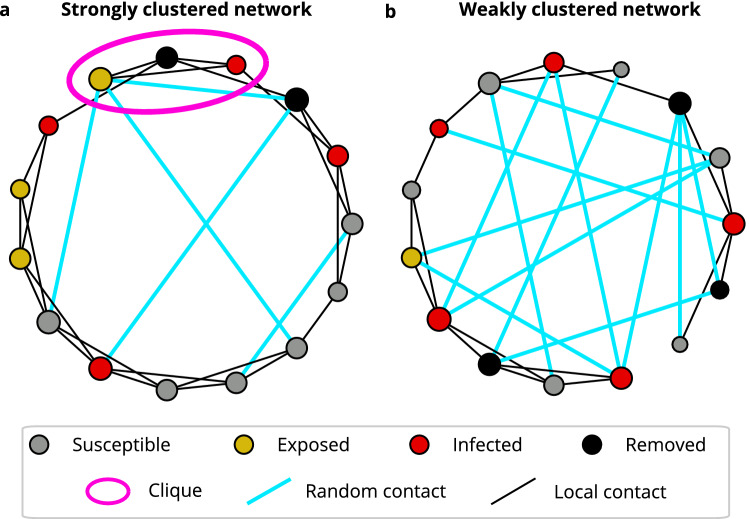
Watts–Strogatz small world network. Agents are placed in a ring-like topology and linked to their *k* nearest neighbors (black lines). Next, every link is rewired randomly with a small probability *p* (cyan lines). Every agent has one of four states: susceptible (gray), exposed (gold), infectious (red), or removed (black). Infectious agents spread the disease to connected susceptible agents with a probability $p_I$ in each time step. The size of network nodes is proportional to the node degree. (**a**) Strongly clustered network. Almost all contacts are restricted to neighbors ($p = 0.1$). (**b**) Weakly clustered network with a large fraction of random contacts ($p=0.5$). Other parameters are equal in (**a**) and (**b**): $n = 15$ agents, $k = 4$ average contacts.

However, while previous works on network-based models for COVID-19 focus on qualitative aspects of certain network ensembles, like power-law growth^19,23,25^, we here aim to explicitly infer the time-dependent transmission network for COVID-19 in Germany. We argue that during the period of severe NPIs, like contact restrictions, the most important feature of real transmission networks is their strong clustering. This means that because public places and events are closed, we expect that people focus their contacts on a single group (clique), where almost each member of this clique is contact with each other. Typical examples of such cliques include households or teams at work. On the other hand, we will neglect the scale-free property of social networks, because it requires that there are a few people with a very large number of contacts, for example at events, schools, large private gatherings etc., which are the targets of most NPIs. We combine Bayesian parameter inference^27^ with an epidemiological model based on the Watts–Strogatz small-world network^17^ that allows to interpolate between unclustered and highly clustered transmission networks by varying the fraction of random contacts in the population, see Fig. 2, to infer the topology of the transmission network in Germany during three time periods in 2020: February 26 until March 15, before serious NPIs were imposed, March 16 until June 6, when strict contact restrictions were in place and nonessential businesses were closed, and June 7 until September 15, when most NPIs were lifted. Random contacts often span a large distance in the transmission network and connect different cliques. They include, for example, contacts in public transport, bars and restaurants, but also contacts with relatives that live far away. Furthermore, given the nature of random contacts, the probability of superspreading events increases when there is a high density of such random contacts, as they enable the disease to spread to fully susceptible cliques.

After inferring the model parameters, we perform a parameter scan to identify the transition points between the linear and the exponential regime of the dynamics. This allows us to associate the three time periods with regions in the phase diagram. Next, we derive a mean-field analysis of a simplified model to gain a deeper understanding of the dynamics in the linear regime. We end with a critical discussion of our results.

## Results

### Bayesian parameter inference

We aimed to infer the induced changes in the topology of the COVID-19 transmission network by Bayesian parameter inference. We expected that NPIs lead to a change in the behavior of people and thus in the topology of the corresponding transmission network. We assumed that the transmission network can be described by the Watts–Strogatz network^17^ that can interpolate between a weakly and a strongly clustered small-world network, see Fig. 2. Crucially, in this framework, we could distinguish local, clustered contacts within cliques, like households, nursing homes, businesses, etc. and random contacts outside of these clusters, corresponding to encounters in public transport, with business partners, friends and family that live far away and similar. During the construction of the Watts–Strogatz network, *n* nodes are placed in a ring topology and connected to their $k=2,4,6,\dots$ nearest neighbors (local contacts, black lines in Fig. 2). After that, each link is rewired with a probability *p* to another random node (random contacts, cyan lines in Fig. 2). We used the SEIR (susceptible-exposed-infectious-removed) epidemiological model for the COVID-19 disease dynamics. Agents in the SEIR model were represented by network nodes such that infectious agents could spread the disease with probability $p_I$ to the susceptible agents that they are connected to in discrete time steps of single days (“Methods” section, Fig. 2, Supplementary Fig. 1).

We inferred the model parameters $p, k, p_I$ for the time periods before and after the first NPIs were implemented in Germany, and after most NPIs were lifted again using an approximate Bayesian computation with sequential Monte Carlo (ABC-SMC) algorithm (“Methods” section). See Table 1 for an overview of the model parameters. We did not infer the mean time periods agents spend in the exposed and infectious states, because they were reported in the literature as $3\hbox {d}\pm 1\hbox {d}$ (mean ± standard deviation) from exposure to becoming infectious and $10\hbox {d}\pm 3\hbox {d}$ of being infectious^29^. We kept the number of agents fixed at $n = 3 \times 10^5$, which we regard as a representative sample of the whole population. Note that we do not explicitly account for the quarantine of infectious agents, which could be done by a time-dependent removal probability, for example. However, we do not do this, because the infection and removal probabilities cannot be determined independently^3^ and there is no reliable data on the effectiveness of TTI measures to further specify the removal probability.

For the time periods before June 6, for which a large number of infections was undetected, we also inferred the initial numbers of exposed and infectious individuals $n_E(0), n_I(0)$.

Based on Google mobility data and previous work on the inference of change points in the spread of COVID-19^3,30^ we assumed the critical time point for the effect of NPIs in Germany to be March 15, as from March 16, NPIs were synchronized in German states, and schools and nonessential businesses were closed.

**Figure 3:**
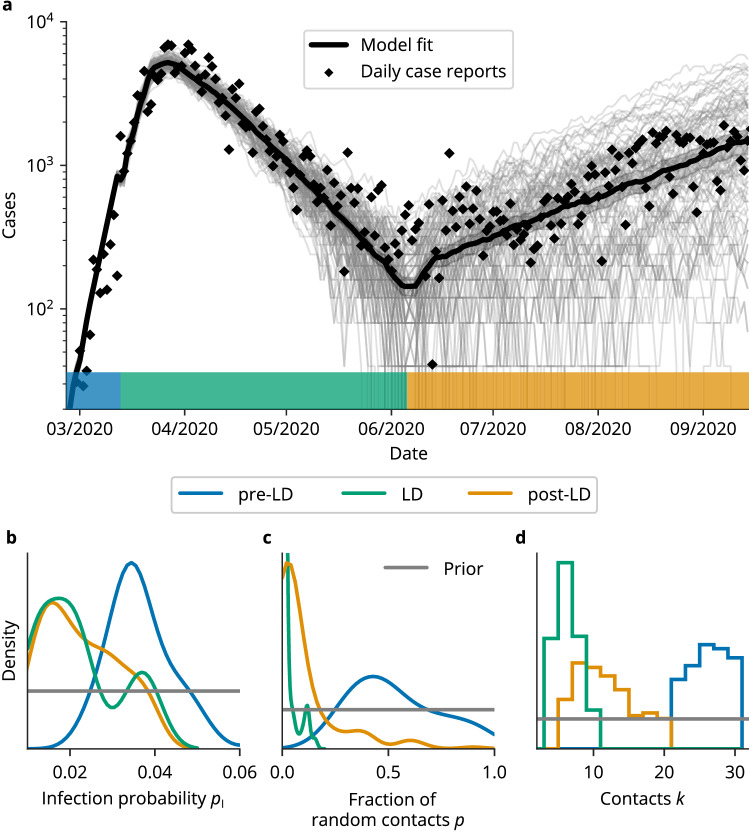
Inference of key epidemiological parameters before (pre-LD, blue), during (LD, green) and after (post-LD, yellow) the lockdown in Germany. (**a**) Daily case reports (black diamonds), single model instances (gray lines) and their mean (black line, error band corresponds to 95% error of the mean). Colors indicate the respective time periods, where the color is the same as that of the corresponding posterior parameter distributions in (**b–d**). Model parameters were chosen as the median of the respective posterior distribution. (**b–d**) Kernel density estimates of the posterior parameter distributions. The uniform prior distribution is shown in gray. (**b**) The infection probability $p_I$ decreased from $p_I = 0.035$ (CI [0.025, 0.061]) pre-lockdown to $p_I = 0.02$ (CI [0.01, 0.04]) during the lockdown. After most restrictions were lifted, the infection probability remained almost unchanged at $p_I = 0.02$ (CI [0.01, 0.04]). (**c**) The fraction of random contacts in the transmission network *p* decreased strongly from $p = 0.48$ (CI [0.23, 0.94]) to $p = 8 \times 10^{-5}$ (CI $[10^{-7}, 0.12]$) when restrictions were put in place and increased to $p = 0.03$ (CI [0.001, 0.60]) when they were lifted. (**d**) The total number of contacts *k* decreased strongly from $k = 26$ (CI [22, 32]) to $k = 6$ (CI [4, 10]) during lockdown before increasing again to $k = 12$ (CI [6, 20]) after restrictions were lifted.

We intentionally chose broad, uninformative priors for all parameters, such that we could compare the obtained posterior distributions with other data sources as a sanity-check of our approach. To account for the weekday-dependent reporting delay, we used a seven-day rolling average of new case reports provided by Johns Hopkins University^28^, see Fig. 3a. Our parameter inference scheme is based on a minimization of the difference between this average and the number of agents becoming infectious in the model on the corresponding day. For the initial phase, we base our inference on the absolute number of infections, while we used the relative number for the following time periods (Supplementary Information).

First, we inferred the parameters for the time from February 26 to March 15, since daily new cases increased rapidly after February 26, while there were almost no cases in the week before. Our analysis of this period revealed an almost random transmission network, with a median fraction of random contacts of $p = 0.48$ (with 95% credibility interval, CI [0.23, 0.94]), a large number of contacts $k = 26$ (CI [22, 32]) and a high infection probability of $p_I = 0.035$ (CI [0.025, 0.061]). The inference of this unexpectedly high number of contacts could be the result of a scale-free degree distribution before NPIs were imposed (Discussion). For the initial condition we estimated that 33 (CI [4, 46]) people were exposed and 81 (CI [32, 118]) people were infectious on February 26.

To assess the changes of the transmission network induced by the NPIs in Germany, we next considered the time period following March 16. During that time frame, the NPIs were changed several times, however, the contact restrictions, which we regard as the most crucial intervention, were only lifted on June 6, which is why we chose this date as the endpoint of the time interval. As the total number of cases was computationally intractable, we here used the relative number of new cases as input for this time period (Supplementary Information).

Our Bayesian parameter inference revealed that the NPIs reduced the number of contacts in the transmission network considerably to $k = 6$ (CI [4, 10]). They also reduced the infection probability of these contacts to $p_I = 0.02$ (CI [0.010, 0.039]), which matches well with an estimation based on the individual-level secondary attack rate in the household of 17%^31^. Crucially, the fraction of random contacts decreased to $p = 7\times 10^{-5}$ (CI $[10^{-7}, 0.12]$), stopping the exponential growth. Additionally, we estimated the number of exposed people on March 16 to be $n_E(\mathrm {March 16}) = 78$ (CI [31, 147]) per million and the number of infectious people to be $n_I(\mathrm {March 16}) = 452$ (CI [301, 656]) per million. The fact that during the week before March 16 there were only 57 infections per million detected in Germany is a hint that a large fraction of infections went unnoticed at the time, which agrees with other reports^32,33^.

We also inferred the model parameters for the time period following June 6 when contact restrictions were lifted. To this end, we used the final time point of simulation instances from the previous period as initial conditions. For the number of contacts, we obtained a median $k = 12$ (CI [6, 20]) that matches well with reports of the average number of daily contacts in Europe of 13.4^34^. The median fraction of random contacts was estimated as $p = 0.03$ (CI [0.001, 0.6]), which means it was notably smaller than before NPIs had been implemented, but larger than in the time period of strict NPIs. Interestingly, we found that the infection probability was as low in this time period at $p_I = 0.02$ (CI [0.01, 0.04]) as during the lockdown, which could be the result of a seasonal effect and of people spending more time outside, which hinders the spread of airborne diseases such as COVID-19. We inferred rather broad parameter posterior distributions for this period, due to the generally low number of infections and large localized outbreaks, leading to a large variation in daily case counts. This is also reflected in a large variability between single model instances for this time period, see Fig. 3a (gray lines). For the time periods without strict NPIs (February 26 to March 15 and June 6 to September 15), for which we inferred a large fraction of random contacts, the infection probability $p_I$ and the number of contacts *k* were notably correlated, leading to broader posterior distributions. Fig. 3 shows the model fit compared to the daily case counts and the corresponding prior and posterior parameter distributions of the network parameters and the infection probability for the three time periods.

### Disease dynamics in the clustered network

**Figure 4:**
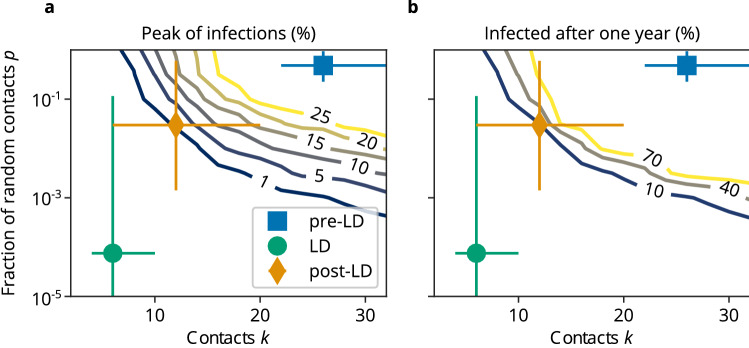
Model dynamics in dependence on the total number of contacts *k* and the fraction of random contacts *p*. (**a**) Number of simultaneously infected people (peak of infections) in percent of population. The wave peak can be massively mitigated by decreasing the fraction of random contacts, even while keeping the total number of contacts constant. (**b**) Cumulative number of infections after one year, in percent of the population. Similar to the peak of infections, the cumulative number of infections can be limited by reducing the fraction of random contacts. Blue square (pre-LD), green point (LD) and yellow diamond (post-LD) correspond to the median parameters and 95% CI obtained from Bayesian parameter inference for the time periods 26/02–15/03, 16/03–05/06 and 06/06–15/09, respectively, and are shown as a reference. The NPIs after March 15 prevented an exponential spread of the disease, but lifting them led to another exponential increase. Shown are contour lines of the mean of 20 independent model realizations of each parameter combination (*p*, *k*), while the other parameters were fixed at $n=10^5, p_I = 0.02$.

To determine the transitions between the linear and exponential regimes of the dynamics, we performed a parameter scan varying the network parameters *p* and *k*, while keeping the disease-specific parameters and the system size fixed at $n = 10^5, p_I = 0.02$. Thereby, reducing *k* corresponds to reducing the total number of contacts (local and random), while reducing *p* does not change the number of contacts, but restricts them to locally clustered agents (cliques). We recorded the peak number of simultaneously infected people, because a central goal of NPIs is to prevent the overload of the health system, and the total number of infected people after 100 days as a measure for the total damage to public health, see Fig. 4.

Importantly, the number of infections could be reduced massively by only decreasing the fraction of random contacts in the population, while keeping the total number of contacts constant. As we have inferred the parameters of the transmission network for different time periods, we could associate them with regions in our parameter space. If no NPIs had been implemented and people would not have changed their behavior, more than 40% of people could have been infected simultaneously and almost everybody would have been infected after one year (Fig. 4, blue square). The peak of infections was reduced to 0.0002% by the interventions (Fig. 4a, green point). Lifting the NPIs moved the system back into the exponential regime, with a projected peak of infections of 1.3% and almost 10% of the population to be infected within one year (Fig. 4, yellow diamond). Reducing the total number of contacts can in principle push the system below the epidemiological threshold leading to extinction of the disease (see Figs. 1, 4), however the effect is weaker in the regime far away from the threshold (see Figs. 1, 4, $k = 10, 12, 14$). On the other hand, in the strongly clustered regime $p \approx 0$, increasing the fraction of random contacts has a dramatic effect: both the peak value of infected agents and the total number of infected agents increase in a non-linear manner. Preventing most random contacts in the network ($p\rightarrow 0$) hinders the spread of the disease, so that the effective reproduction number fluctuates around 1, and the cumulative number of infections increases linearly with time as observed in many countries after the first NPIs were imposed.

### Wave speed of infections in the linear regime

We were especially interested in the disease dynamics in the regime $p \rightarrow 0$, as this is where traditional epidemiological models that assume a random transmission network break down. In the case of only local contacts in the network, the disease spreads like a wave originating from the initially infectious agent. This wave-like disease spread was also reported in real networks, such as the air traffic network^35^. To calculate the speed of the infection wave, we used a mean-field approximation of an SIR-like agent-based model operating on the Watts–Strogatz network (Supplementary Information). We scaled the mean-field equations to continuous time *t* and space *x* (where the distance $\Delta x$ is measured as the number of links along the ring of nodes), and approximated the dynamics by a set of partial differential equations for the probability densities of susceptible $\sigma (x,t)$, infectious $\iota (x,t)$ and removed $\rho (x,t)$ agents (Supplementary Information). In particular, we obtained the following equation for the probability density of infectious agents

1 $$\begin{aligned} \partial _{tt} \iota (x,t) = \frac{2}{\tau } \left\{ -\partial _t \iota (x, t) +\kappa _I k \sigma (x,t) \left[ (1-p) \left( \iota (x,t) \right) + p I(t) \right] - \kappa _R \iota (x,t) \right\} + \kappa _I k (1-p) D_k \sigma (x,t) \partial _{xx} \iota (x,t), \end{aligned}$$

where $\kappa _I$ is the infection rate, and $\kappa _R$ is the removal rate. Here, $\tau$ is the short time scale of the local disease dynamics of a single agent, while $D_k := \frac{\Delta x^2 {\tilde{k}}}{\tau }$, with ${\tilde{k}} := (k/2 + 1)(k + 1) / 12$ is a constant that determines the disease spread in the network on longer time scales. The total number of infectious agents *I* is defined as $I := \int \iota \, {\mathrm {d}}x$, where the integral represents a nonlocal coupling by random contacts. The equation resembles the Telegrapher’s equation but with a nonlocal coupling by random contacts and a nonlinear diffusion term due to local contacts. We recover the classical SIR model for $p = 1$, as expected. For the regime $p=0$, we obtained the speed of disease spread through the network as

2 $$\begin{aligned} c = \sqrt{\kappa _I k D_k} \propto k\sqrt{k}. \end{aligned}$$

**Figure 5:**
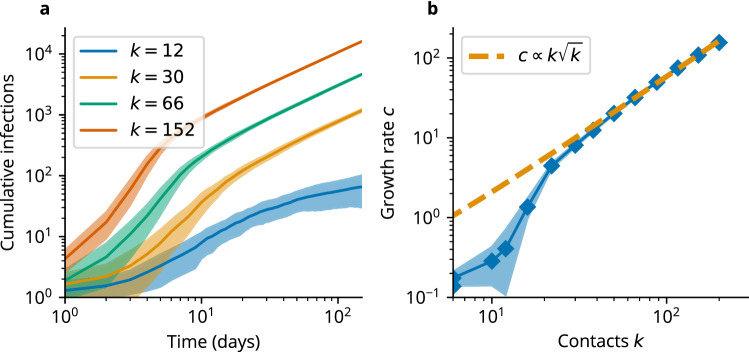
Comparison of model dynamics and mean-field approximation in the linear regime. (**a**) Cumulative number of infections in the model for a highly clustered network ($p=0$). (**b**) Growth rate of cumulative infections in dependence of the number of contacts *k*. The growth rate scales as $c\propto k \sqrt{k}$ as predicted by mean-field theory for a large number of contacts *k*, see Eq. (2). Shown is the mean and standard error of the mean (SEM) of 20 independent simulations for each parameter.

Notably, the wave speed depends on *k* in a nonlinear manner. Comparing our prediction against simulation data (Supplementary Information) revealed that the wave speed is proportional to the growth rate of the cumulative infections in the linear regime, see Fig. 5.

## Conclusion

We used Bayesian parameter inference to quantify the effects of government interventions in Germany on the transmission network of COVID-19 assuming it can be approximated by a Watts–Strogatz network. This network captures the key feature of social networks affected by NPIs, namely the strong clustering of contacts when people restrict their social life to a small, interconnected clique. Our analysis revealed that NPIs lead to a reduction in transmission probability, number of contacts, and, crucially, to the removal of almost all contacts outside highly clustered cliques. In contrast to standard epidemiological models, in this regime the cumulative number of infections does not increase exponentially but linearly, with a massively reduced peak of infections. The dynamics corresponds to a wave-like spread of the disease in the network, whose wave speed *c* we predicted by mean-field theory to scale as $c \propto k \sqrt{k}$ in dependence of the number of contacts *k*. At the same time, the effective reproduction number fluctuates around 1, irrespective of the wave speed. However, as long as the epidemic threshold is not reached by the reduction of contacts between cliques and the reduction of the infection probability, the disease still spreads in the population, which emphasizes the need for an effective test, trace, and isolate (TTI) system and a vaccinations.

Several other studies have highlighted the importance of random contacts between members of different cliques. A study that used mobility network data to show that the spread of the disease is mostly driven by infections at events which connect different communities, for example in restaurants and religious establishments, further supports the importance of random contacts^36^. Moreover, empirical studies of the circumstances under which people got infected revealed that although 46% to 66% of transmission is household-based (clustered contacts), random contacts between these cliques are essential to sustain the epidemic, even if they only cause a low percentage of infections directly^37^. A theoretical study that investigated the effect of different social network-based distancing strategies showed that the most effective social distancing strategy is to restrict contacts to a single clique, and to eliminate any contacts between the cliques (random contacts)^38^.

We based our parameter inference on the daily count of positive tests. This approach is not perfect: the number of positive tests depends on the testing policy and the number of available tests. Also, the date, when the test result is recorded is always delayed. Another option is the usage of daily deaths, which are independent of testing. However, it is very difficult to base our approach on the death count, because the total death count in Germany during the first wave and especially during the summer was comparatively low. That means that even our scaled-down system would be intractably large, if we want to reproduce the number of deaths using realistic assumptions about the fatality rate of the disease. Additionally, the time distribution between infection and death has a large variance of up to 10 days, as it varies depending on the age of the affected individual^29,39^, increasing the noise in the already low death numbers. We further assumed that all people that were tested positive were also infectious at the time of the test and that the time delay between people turning infectious and their test is negligible. In future studies, the time delay between testing and the onset of infectiousness could be accounted for using nowcasting^40^.

In our model we assumed a Watts–Strogatz transmission network and distinguished between random and clustered links. It is possible to use other algorithms to construct clustered networks, for example the stochastic block model^41^, the relaxed caveman graph^42^, or the configuration model with defined clustering^43^, to name a few. We chose the Watts–Strogatz graph for its simplicity and its low number of parameters, but we expect that other graph ensembles would yield similar results, as long as they allow for high clustering and overlap of cliques. A potential target of future research could be the identification of the most appropriate network ensemble for different lockdown scenarios.

We did not account for the scale-free degree distribution found in real social networks. It is a result of hubs with a large number of connections potentially connecting several communities, like grocery stores, restaurants, religious establishments, etc. This favors disease spread even more than randomly assigned links and can lead to outbursts of infections (superspreading events)^26^. In fact, there were several superspreading events related to carnival festivities at the beginning of the first wave in Germany^32^. We believe that this, at least in part, explains why our parameter inference scheme finds larger values for the total number of social contacts *k* than previously reported^34^ for the time period before NPIs were put in place. However, the fact that our inferred parameters for the later time periods are consistent with other reports regarding the number of contacts and the infection probability, reassures us that our assumption, that super spreading was less important after NPIs were enacted, is justified.

We inferred that lifting contact restrictions in Germany after June 6 moved the disease dynamics back into the exponential regime. However, the effective reproduction number remained close to one, due to a reduced transmission probability of the disease, compared to the pre-lockdown time period. This is likely a result of several phenomena: first, there is probably a small seasonal reduction in virus transmission due to the higher temperatures when no lockdown policy is in effect^44^. Second, even after contact restrictions were lifted, there was now a mask mandate at public places, which has been shown to reduce the daily growth rate of new cases by around 47% in Germany^45^. Lastly, mobility was still reduced by about 20% compared to previous years^30^ correlating with lower virus transmission and indicating a higher awareness of the virus in the population^46,47^. In October, people did spend more time indoors again, and mobility reduced to normal levels, contributing to a second fast exponential growth of cases. The German government responded with new NPIs in November, which reduced the effective reproduction number to a value around one, again corresponding to a linear regime of new cases.

Our analysis shows that NPIs can reduce the effective reproduction number to one by eliminating random contacts. However, eliminating the disease does most likely require to cut almost all contacts between different cliques, for example, by working from home. In summary, government interventions should target random contacts and encourage people to restrict their contacts to a single clique in order to efficiently prevent disease spread.

## Methods

### Model definition

We study an agent-based, discrete-time SEIR model on the classical Watts–Strogatz network, representing agents as network nodes. The network is constructed by, first, connecting every node to its *k* nearest neighbors in a ring-like topology, and, second, rewiring every link to a random node with probability *p*, see Fig. 2. Every node *i* has a discrete state $s_i \in {\mathcal {S}} = \{S, E, I, R \}$, corresponding to susceptible (*S*), exposed (*E*), infectious (*I*), and removed (*R*) states.

Disease progression is dictated by $\Gamma$-distributed waiting times inferred from COVID-19 disease characteristics, as these have been found to describe the disease progression best^29^. During every discrete time step *t*, where the length of the time step corresponds to one day, each susceptible agent can become exposed with probability $p_I$ for every infectious agent they are connected to,

3 $$\begin{aligned} P\left( s_i(t+1) = E|s_i(t) = S\right) = 1 - (1-p_I)^{I_i}, \end{aligned}$$

where $I_i$ is the number of infectious agents node *i* is connected to. Upon infection, we change the agent’s state to exposed, and assign a waiting time $\tau _E \sim \Gamma (k_E, \theta _E)$ which we draw from a $\Gamma$-distribution with shape $k_E$ and scale $\theta _E$. In every time step, the waiting times are reduced by one day, $\tau _E(t+1) = \tau _E(t) - 1$ if $\tau _E(t) > 0$. Else, the disease progresses, $s_i(t+1) = I$, and a new waiting time is assigned from another $\Gamma$-distribution $\tau _I \sim \Gamma (k_I, \theta _I)$, with shape $k_I$ and scale $\theta _I$. Finally, when $\tau _I \le 0$, the node is removed, $s_i(t+1) = R$. A sketch of the SEIR dynamics can be found in Supplementary Fig. 1. See Table 1 for an overview of all model parameters.

**Table 1:** Model parameters.

Symbol	Description
*k*	Mean number of contacts
*p*	Fraction of random contacts
$p_I$	Infection probability
$\left\langle \tau _{E,I}\right\rangle$	Mean exposed/infectious duration
$\left\langle \Delta \tau _{E,I}^2 \right\rangle$	Variance of exposed/infectious duration

We calculate the shape and scale of the $\Gamma$-distributions from the reported mean and variance of the time in the respective states according to

4 $$\begin{aligned} k_{E,I}= & {} \frac{\left\langle \tau _{E,I}\right\rangle ^2}{\left\langle \Delta \tau _{E,I}^2 \right\rangle } , \end{aligned}$$

5 $$\begin{aligned} \theta _{E,I}= & {} \frac{\left\langle \Delta \tau _{E,I}^2 \right\rangle }{\left\langle \tau _{E,I}\right\rangle }. \end{aligned}$$

Note that we did not infer these parameters with our Bayesian parameter inference framework.

In our model we do not account for an inflow of infectious people by travel; we instead account for the initial surge of infections by placing randomly $n_{E}(0)$ exposed and $n_{I}(0)$ infectious agents in the population.

### Bayesian parameter inference

We apply approximate Bayesian computation with a sequential Monte–Carlo scheme (ABC-SMC) to infer the set of parameters $\Theta = \{ p_I, p, k, n_{E}(0), n_{I}(0) \}$ of our agent-based model. We always keep the total number of agents fixed at $n = 3 \cdot 10^5$. To this end, we employ the Python package pyABC^48^. In short, the algorithm employs sequential importance sampling over generations $T=1,...,n_T$. In generation *T*, the algorithm draws sets of parameters $\theta _i$ from a given proposal distribution and consequently simulates data $C^{(i)}$ from the model, until $n_{\mathrm{ABC}}$ instances were accepted based on the comparison to observed data via a distance function $D(C^{(i)}, C_{\mathrm {obs}})$ and acceptance threshold $\varepsilon _T$, $D(C^{(i)},C_{\mathrm {obs}})\le \varepsilon _T$. As the distance function, we choose the absolute difference between new cases in the model instance $C^{(i)}(t)$ and the respective reports for Germany $C_{\mathrm {obs}}(t)$,

6 $$\begin{aligned} D(C^{(i)}, C_{\mathrm {obs}}) := \sum _t \left| C^{(i)}(t) - C_{\mathrm {obs}}(t) \right| . \end{aligned}$$

New cases in the model are given by the daily new infections

7 $$\begin{aligned} C^{(i)}(t) = n_I^{(i)}(t) - n_I^{(i)}(t-1) + n_R^{(i)}(t) - n_R^{(i)}(t-1). \end{aligned}$$

They are compared to the 7-day rolling average of new case reports in Germany $C_{\mathrm {obs}}(t)$ provided by Johns Hopkins University^28^ to account for the weekly fluctuations in reporting. Acceptance of model instances depends on the acceptance threshold $\epsilon _T$ of generation *T*, which we choose as the median of the distances of the accepted instances of the previous generation

8 $$\begin{aligned} \epsilon _{T+1} = {\mathrm {median}}\left( \left\{ D(C^{(i)}, C_{\mathrm {obs}}) < \epsilon _T \right\} \right) . \end{aligned}$$

When $n_{\mathrm {ABC}}$ model instances have been accepted, the algorithm constructs new proposal distributions from the accepted instances to allow high acceptance rates while decreasing the threshold^49^. In particular, for the continuous variables it employs a multivariate normal distribution with an adaptive covariance matrix based on the sample covariance matrix, whose scale parameter is determined by a grid search with fivefold cross validation and refitting on the whole data set. We compute the discrete numbers of initially exposed and infectious people $n_{E}(0), n_{I}(0)$ by rounding the continuous output of the multivariate normal distribution. We can do this without a large error as these parameters vary smoothly over a large range. For the parameter *k* we employ an adaptive discrete transition that assigns probabilities to all possible parameter values directly from the frequency of the respective value in the population of accepted particles with additional random jumps (with probability 0.3) to ensure absolute continuity of the prior. The process is repeated until the acceptance threshold is sufficiently low; we especially ensured that the threshold is considerably lower than the difference between the reported cases and the seven-day rolling average of cases.

## Supplementary Information

Supplementary Information.

## Data Availability

The simulation code and input data are available as Zenodo snapshot at https://doi.org/10.5281/zenodo.4884171.
