# Supplementary material for: Inferring the effect of interventions on COVID-19 transmission networks
Source: Sci Rep. 2021 Nov 9;11:21913. doi: 10.1038/s41598-021-01407-y (PMC8578219; doi:10.1038/s41598-021-01407-y)
Supplement: Supplementary file 1 — Supplementary Information. [file 41598_2021_1407_MOESM1_ESM.pdf]

Supplementary material for: Inferring the effect of interventions on COVID-19 transmission networks

Simon Syga[1], Diana David-Rus[2], Yannik Schälte[3,4], Haralampos Hatzikirou[1,5], Andreas Deutsch[1,*]

1 Center for Information Services and High Performance Computing, Technische Universität Dresden, Nöthnitzer Straße 46, 01062 Dresden, Germany
2 Bavarian Health and Food Safety State Authority (LGL) – Research division, Veterinärstraße 2, 85764, Oberschleißheim, Germany
3 Institute of Computational Biology, Helmholtz Zentrum München – German Research Center for Environmental Health, 85764 Neuherberg, Germany
4 Center for Mathematics, Technische Universität München, 85748 Garching, Germany
5 Mathematics Department, Khalifa University, P.O. Box 127788, Abu Dhabi, United Arab Emirates

*andreas.deutsch@tu-dresden.de

1 Bayesian parameter inference

February 26 - March 15

The early phase of the epidemic is characterized by a low number of cumulative infections. We can therefore directly use the absolute numbers of new infections as input for our agent-based model, as we are always far away from the epidemic threshold. We choose broad, uniform priors for all the parameters that can be found in table S1. Note that the probability for random connections varies on several orders of magnitude $p \in [10^{-6}, 10^{0}]$ and we therefore infer this parameter on a logarithmic scale. We use $n_{\mathrm{ABC}} = 100$ and obtain an effective sample size of $n_{\mathrm{eff}} \approx 46$.

Table S1. Priors of model parameters for the time period February 26 to March 15.

Parameter	Variable	Prior distribution
Infection probability	p_I	`Uniform(0.01,0.07)`
Probability of random links	$\log_{10} p$	`Uniform(0,-6)`
Number of links	$k/2$	`DiscreteUniform(1,16)`
Initially exposed	$n_E(0)$	`Uniform(0,47)`
Initially infectious	$n_I(0)$	`Uniform(0,160)`

March 16 - June 6

In the time period from March 16 to June 6 Germany recorded 177652 cases in total. This means that it becomes computationally unfeasible to replicate the population directly in our model without noticing a strong effect of the removed (immune) agents. Therefore, we scale down the total number of infections to our system size and compare the relative number of cases per 300,000 people instead. Assuming our hypothesis that the NPIs lead to a strongly clustered transmission network holds, we expect a large number of unconnected communities in Germany in that time period, which we represent as distinct model instances. We use $n_{\mathrm{ABC}} = 200$ and obtain an effective sample size of $n_{\mathrm{eff}} \approx 105$. Our priors for this period can be found in table S2.

June 7 - September 15

To infer the parameters of the system during this time period we first sample parameters from the posterior distribution which we obtained for the previous time period, and let the system evolve for 81 days (corresponding to the time period from March 16 to June 6). Next, we change the infection probability p_I and

Table S2. Priors of model parameters for the time period March 16 to June 6.

Parameter	Variable	Prior distribution
Infection probability	p_I	`Uniform(0.01,0.03)`
Probability of random links	$\log_{10} p$	`Uniform(0,-6)`
Number of links	$k/2$	`DiscreteUniform(1,11)`
Initially exposed	$n_E(0)$	`Uniform(3,57)`
Initially infectious	$n_I(0)$	`Uniform(38,414)`

assign a new transmission network based on a new set of parameters p, k. For these parameters, we choose the same prior distributions as for the previous time period, see Table S3. We use $n_{\text{ABC}} = 200$ and obtain an effective sample size of $n_{\text{eff}} \approx 164$.

Table S3. Priors of model parameters for the time period June 7 to September 15.

Parameter	Variable	Prior distribution
Infection probability	p_I	`Uniform(0.01,0.03)`
Probability of random links	$\log_{10} p$	`Uniform(0,-6)`
Number of links	$k/2$	`DiscreteUniform(1,11)`

2 Parameter scan

SEIR model

To investigate the disease dynamics in the small-world network, we perform a parameter scan. We vary the network parameters p, k while keeping the rest of the parameters fixed at $n = 10^5, p_I = 0.02, n_E(0) = 0, n_I(0) = 10$. Our choice for p_I during the parameter scan is motivated by reports of the COVID-19 individual-level secondary attack rate (SAR) in the household of 17 %. Inverting Eq 3 we obtain

$$p_I = 1 - \sqrt[\tau]{1 - SAR} \approx 0.02. \tag{S1}$$

We vary the number of contacts from $k = 2, 4, \ldots, 24$ and sample the probability for random contacts in eleven equally-spaced steps on the log scale from $\log_{10} p = -5, \ldots, 0$. As initial condition, 10 random agents are set to the infectious state and the system is simulated until there are no more exposed and infectious agents. We repeat this process five times per parameter combination (p, k). As output we determine the peak of simultaneously infectious people

$$n_{\text{peak}}(p, k) := \max_t n_I(t, p, k), \tag{S2}$$

and the cumulative infection curves

$$N(t, p, k) = n_I(t, p, k) + n_R(t, p, k). \tag{S3}$$

SIR model

To compare our analytical prediction of the wave speed in the highly clustered network (see below), we define a simplified SIR model. The difference to the SEIR model is that there is no exposed state, and that the waiting times for the transition from the infectious to the removed state are drawn from an exponential distribution with mean $\langle \tau_I \rangle = 10d$. For the parameter scan, we initialze the system of $n = 10^5$ agents in a ring-like topology (no random links) with a single infectious agent and let it evolve for 365 time steps. We repeat this process 20 times per parameter k. We then record the cumulative infection curves

$$N(t, k) = n_I(t, k) + n_R(t, k), \tag{S4}$$

see Fig. 5**a**. We calculate the linear growth rate from the cumulative infections as

$$c(k) := \left\langle \frac{N(t_{\max,k},k) - N(t_{\min},k)}{t_{\max,k} - t_{\min}} \right\rangle, \tag{S5}$$

where we neglect the initial exponential growth by skipping $t_{\min} = \langle \tau_I \rangle = 10$ time steps. We also determine the maximum time $t_{\max,k}$ until the epidemic dies out for each parameter k so that after $t_{\max,k}$ time steps, the cumulative number of infections did not increase in any realization of the system

3 Mathematical analysis

Epidemic threshold

We can calculate an upper bound for the number of contacts k_c by demanding $R_0 = 1$, i. e. a single infectious person in a network of susceptible people will effect on average one other person. Using Eq 3 (Main Text) we can calculate the expected number of infections as

$$R_0 = 1 = k_c \left[1 - (1 - p_I)^{\tau_I}\right] \Longleftrightarrow k_c = \frac{1}{1 - (1 - p_I)^{\tau_I}}. \tag{S6}$$

For $p_I = 0.02$ and $\langle \tau_I \rangle \approx 10$ we obtain $k_c \approx 5.5$.

Derivation of differential-equation approximation

In order to predict the linear growth of infections in the highly clustered small-world network, we consider a simplified variant of our original model, where we neglect the exposed state and assume that the progression times are distributed exponentially (SIR model, section 2.2 in Supplementary Information). Then we describe the state of an agent j in our model by a set of three Boolean stochastic variables $s_j(t), i_j(t), r_j(t) = 0, 1$, where $s_j + i_j + r_j = 1$ and $s_j = 1$ indicates that the agent is susceptible, $i_j = 1$ means he is infectious and if $r_j = 1$ he is removed. We represent the event "agent j becomes infectious at time t" by the Boolean stochastic variable $\alpha_j(t)$ with

$$P\left(\alpha_j(t) = 1\right) = 1 - (1 - p_I)^{I_j(t)}, \tag{S7}$$

where $I_j(t) := \sum_{m \in \mathcal{N}_j} i_m(t)$ is the number of infectious agents in the neighborhood $\mathcal{N}_j$ of agent j. Similarly, the event "agent j is removed at time t" is given by the stochastic variable $\beta_j(t)$ with

$$P\left(\beta_j(t) = 1\right) = p_R = 1/\tau_I. \tag{S8}$$

During one time step the state of all agents changes as

$$s_j(t+1) = s_j(t) - \alpha_j(t) s_j(t), \tag{S9}$$
$$i_j(t+1) = i_j(t) + \alpha_j(t) s_j(t) - \beta_j(t) i_j(t), \tag{S10}$$
$$r_j(t+1) = r_j(t) + \beta_j(t) i_j(t). \tag{S11}$$

We want to calculate the expected value of the state variable under a mean-field approximation, i. e. we replace the expected value of a function $f(X)$ of any random variable X by the function evaluated at the expected value of the random variable, $\langle f(X) \rangle \approx f(\langle X \rangle)$. In particular, this also means that we neglect any correlations between the state variables of neighboring nodes. We denote the expected values of the state variables as $\sigma_j(t) := \langle s_j(t) \rangle, \iota_j(t) := \langle i_j(t) \rangle, \rho_j := \langle r_j(t) \rangle$. For the expected number of infectious neighbors we obtain

$$\langle I_j(t) \rangle = (1 - p) \sum_{m=-k/2}^{k/2} \iota_m(t) + \frac{kp}{N-1} \sum_{m \neq j} \iota_m(t), \tag{S12}$$

where N is the total number of nodes in the network and p is the probability of a random link (see Model definition).

Next, we also introduce a small time step length $\tau > 0$ and continuous time $\hat{t} = \tau t$ along with the transition rates $\kappa_I := p_I/\tau, \kappa_R := p_R/\tau$. In the following we only consider continuous time and drop the hat for better readability. For $\tau \to 0$ we can approximate the expected value of $\alpha_j(t)$ by a Taylor approximation around $p_I = 0$ as

$$P\left(\alpha_j(t) = 1\right) = 1 - (1 - \kappa_I \tau)^{I_j(t)} \approx \kappa_I \tau I_j(t). \tag{S13}$$

We can further simplify our system by considering the regime $N \to \infty$, and introducing the spatial step length $\Delta x > 0$ so that $N\Delta x = L = \text{const}$. We can then replace the state probabilities $\sigma_j(t), \iota_j(t), \rho_j(t)$ by the probability densities $\sigma(x = j\Delta x, t), \iota(x = j\Delta x, t), \rho(x = j\Delta x, t)$. This allows us to approximate the expected number of infectious agents in the neighborhood by expanding the terms $\iota(x + m\Delta x, t) \approx \iota(x, t) + m\Delta x \partial_x \iota(x, t) + m^2 \Delta x^2 / 2 \partial_{xx} \iota(x, t)$ to obtain

$$\begin{aligned} \langle I_j(t) \rangle &= (1-p) \sum_{m=-k/2, m\neq 0}^{k/2} \iota_{j+m}(t) + \frac{kp}{N-1} \sum_{m \neq j} \iota_m(t) \approx \\ &\approx (1-p)k\left(\iota(x,t) + \Delta x^2 \tilde{k} \partial_{xx} \iota(x,t)\right) + \frac{kp}{L} \int_0^L \iota(x,t)\,\mathrm{d}x =: \Psi(x,t), \end{aligned} \tag{S14}$$

where $\tilde{k} := (k/2 + 1)(k+1)/12$. Approximating all time-dependent functions by their Taylor approximation up to second order $f(t+\tau) \approx f(t) + \tau f'(t) + \tau^2/2 f''(t)$ and rearranging terms we obtain the following set of non-linear PDEs for the expected value of the agents' states

$$\partial_{tt}\sigma(x,t) + \frac{2}{\tau}\partial_t \sigma(x,t) = -\frac{2}{\tau}\kappa_I \sigma_j(t) \Psi(x,t), \tag{S15}$$

$$\partial_{tt}\iota(x,t) + \frac{2}{\tau}\partial_t \iota(x,t) = \frac{2}{\tau}\left[\kappa_I \sigma(x,t)\Psi(x,t) - \kappa_R \iota(x,t)\right], \tag{S16}$$

$$\partial_{tt}\rho(x,t) + \frac{2}{\tau}\partial_t \rho(x,t)) = \frac{2}{\tau}\kappa_R \iota(x,t). \tag{S17}$$

We introduce the constant $D_k := \frac{\Delta x^2 \tilde{k}}{\tau}$ and can now identify two separate time scales: A fast time scale, with terms $\propto \frac{1}{\tau}$ which characterizes the disease progression, and a slow timescale with terms $\propto D_k$ that describes the wave of infections in the network. Finally, with $I(t) := 1/L \int_0^L \iota(x,t)\,\mathrm{d}x$ as the total number of infectious people, we obtain the following set of nonlocal PDEs

$$\partial_{tt}\sigma(x,t) = -\frac{2}{\tau}\left\{\partial_t \sigma(x,t) + \kappa_I k \sigma(x,t)\left[(1-p)\left(\iota(x,t)\right) + pI(t)\right]\right\} - \kappa_I k \sigma(x,t) D_k \partial_{xx}\iota(x,t), \tag{S18}$$

$$\begin{aligned} \partial_{tt}\iota(x,t) &= \frac{2}{\tau}\left\{-\partial_t \iota(x,t) + \kappa_I k \sigma(x,t)\left[(1-p)\left(\iota(x,t)\right) + pI(t)\right] - \kappa_R \iota(x,t)\right\} + \\ &\quad + \kappa_I k \sigma(x,t) D_k \partial_{xx}\iota(x,t), \end{aligned} \tag{S19}$$

$$\partial_{tt}\rho(x,t) = \frac{2}{\tau}\left\{-\partial_t \rho(x,t)) + \kappa_R \iota(x,t)\right\}. \tag{S20}$$

In the regime $\tau \to 0$ we expect the the system to quickly reach a local steady state, so that the fast time scale terms vanish. We can use this to obtain an approximate value for the wave speed of infections.

Calculation of the infection wave speed in the highly clustered network

To calculate the wave speed of infections we consider the regime $p = 0$, so that we can neglect the nonlocal coupling by $I(t)$ in Eq S18. As described above, if we let $\lim \tau, \Delta x \to 0$ with finite D_k, we expect the system to always be in a local steady state, corresponding to the fast time scale terms being 0. Then, we arrive at the following equation for the density of infected people

$$\partial_{tt}\iota(x,t) = \kappa_I k D_k \sigma(x,t) \partial_{xx}\iota(x,t). \tag{S21}$$

If we consider a point far away from the wave front, we have $\sigma(x,t) \approx 1$ (everyone is susceptible), and Eq S21 reduces to the wave equation

$$\partial_{tt}\iota(x,t) = \kappa_I k D_k \partial_{xx}\iota(x,t) \tag{S22}$$

with the wave speed $c = \sqrt{\kappa_I k D_k} \propto k\sqrt{k}$.

Supplementary Figures

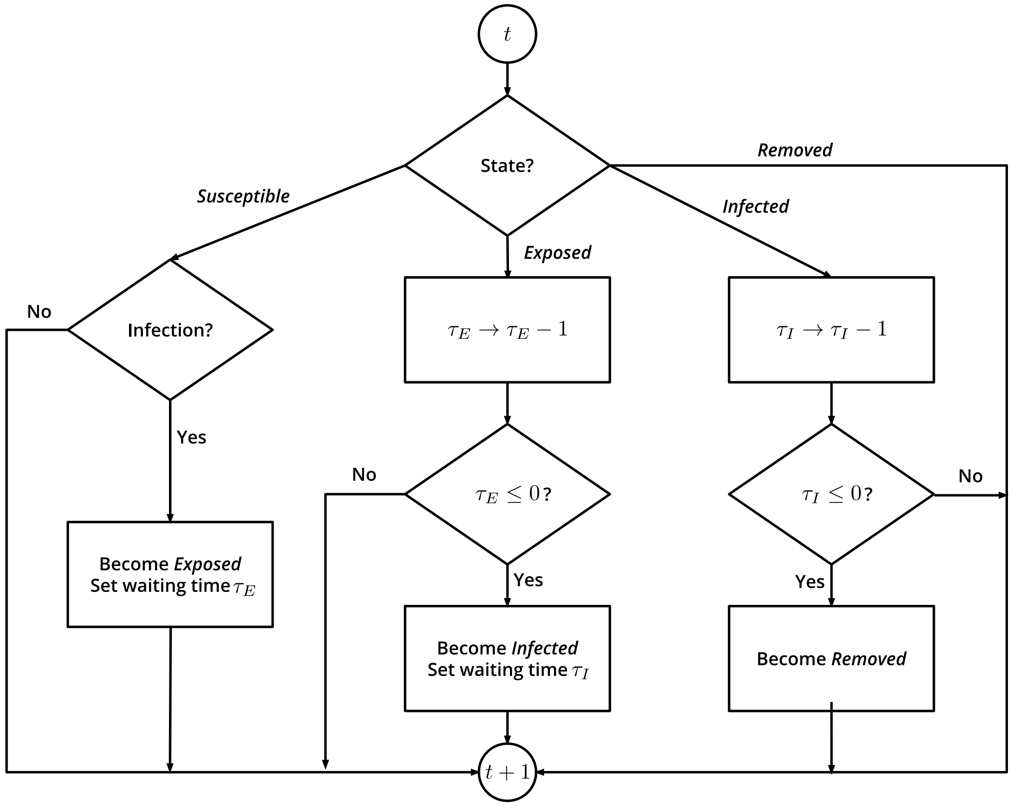

Figure S1. Dynamics in the agent-based SEIR model. Susceptible agents can become exposed, if they are linked to infectious agents. Exposed agents become infectious after the waiting time τ_E, and infectious agents are removed after τ_I. All nodes are updated simultaneously at every time step t.
